# Improvement in health-related quality of life after therapy with omeprazole in patients with coronary artery disease and recurrent angina-like chest pain. A double-blind, placebo-controlled trial of the SF-36 survey

**DOI:** 10.1186/1477-7525-9-77

**Published:** 2011-09-22

**Authors:** Jacek Budzyński, Grzegorz Pulkowski, Karol Suppan, Jacek Fabisiak, Marcin Majer, Maria Kłopocka, Beata Galus-Pulkowska, Marcin Wasielewski

**Affiliations:** 1 University Chair of Gastroenterology, Vascular Diseases and Internal Medicine, Nicolaus Copernicus University in Toruń, Ludwik Rydygier Collegium Medicum, Bydgoszcz, Poland; 2 Clinical Ward of Vascular Diseases and Internal Medicine, Dr Jan Biziel University Hospital No. 2, Bydgoszcz, Poland; 3 Division of Gastroenterological Nursing, University Chair of Nursing and Obstetrics, Nicolaus Copernicus University in Toruń, Ludwik Rydygier Collegium Medicum, Bydgoszcz, Poland; 4 Division of General Practice, Dr Jan Biziel University Hospital No. 2, Bydgoszcz, Poland

**Keywords:** quality of life, SF-36 questionnaire, chest pain, omeprazole, coronary artery disease

## Abstract

**Background:**

Many patients with coronary artery disease (CAD) have overlapping gastroenterological causes of recurrent chest pain, mainly due to gastroesophageal reflux (GER) and aspirin-induced gastrointestinal tract damage. These symptoms can be alleviated by proton pump inhibitors (PPIs). The study addressed whether omeprazole treatment also affects general health-related quality of life (HRQL) in patients with CAD.

**Study:**

48 patients with more than 50% narrowing of the coronary arteries on angiography without clinically overt gastrointestinal symptoms were studied. In a double-blind, placebo-controlled, cross-over study design, patients were randomized to take omeprazole 20 mg bid or a placebo for two weeks, and then crossed over to the other study arm. The SF-36 questionnaire was completed before treatment and again after two weeks of therapy.

**Results:**

Patients treated with omeprazole in comparison to the subjects taking the placebo had significantly greater values for the SF-36 survey (which relates to both physical and mental health), as well as for bodily pain, general health perception, and physical health. In comparison to the baseline values, therapy with omeprazole led to a significant increase in the three summarized health components: total SF-36; physical and mental health; and in the following detailed health concept scores: physical functioning, limitations due to physical health problems, bodily pain and emotional well-being.

**Conclusions:**

A double dose of omeprazole improved the general HRQL in patients with CAD without severe gastrointestinal symptoms more effectively than the placebo.

## Background

Improvement in health-related quality of life (HRQL) is an important purpose of medical interventions. The evaluation of HRQL is also important for measuring quality of care and clinical effectiveness, as well as in assessing reimbursement decisions [1]. HRQL can be assessed using a number of instruments; they may estimate the overall (generic) HRQL, for example through the SF-36 questionnaire, as well as using disease-specific questionnaires such as the following: the Quality of Life in Reflux and Dyspepsia questionnaire (HRQLRAD), the MacNew Heart Disease Quality of Life instrument, and the quality of life questionnaire for patients with atrial fibrillation (AF-HRQL) [2]. The advantage of using a generic HRQL instrument is the possibility of measuring patients' overall state of health (physical, emotional and social), their level of general performance, work productivity loss and a comparison of the outcome of different interventions and clinical conditions through HRQL. The most commonly used generic HRQL instrument is the SF-36 Health Survey, which evaluates eight main health concepts: physical functioning, bodily pain, role limitations due to physical health problems, role limitations due to personal and emotional problems, emotional well-being, social functioning, energy/fatigue (vitality), and general health perception, which can be summarized into physical and mental components [3,4].

Patients with coronary artery disease (CAD) and refractory or recurrent retrosternal symptoms have a reduction in life expectancy and HRQL compared to patients with stable coronary artery disease [4-7]. The causes of this state are frequently the lack of the possibility of revascularization, atherosclerosis progression, instability of the subsequent atherosclerotic plaques, or in-stent restenosis [8], as well as microvascular coronary disease and abnormal cardiac nociception [9]. However, more than 30% of patients with CAD suffer from persistent chest pain which is due to extra-cardiac sources overlapping or mimicking precordial symptoms originating in the heart [10,11]. These are mainly due to the coexistence of gastroesophageal reflux (GER), aspirin-induced gastrointestinal tract damage, and musculoskeletal or panic disorders [4,11-14]. It has been reported that gastrointestinal symptoms have a strong negative influence on the physical, psychological and social functioning in patients with cardiovascular diseases, requiring the use of acetylosalicylic acid and the relief of these symptoms, independently of the kind of therapy, has improved patients' HRQL [4,15].

Proton pump inhibitors (PPIs) or gastric hydrochloric acid secretion inhibitors are used in the treatment of GER, gastric and duodenal ulcer disease, *Helicobacter pylori *eradication, in the prevention of gastric and duodenal damage during therapy with non-steroidal anti-inflammatory drugs, and in empirical therapy in the so-called "omeprazole test", as the first step in the diagnosis of suspected GER-related chest pain [10,11]. In our previous paper, we demonstrated that the double dose of omeprazole (2 × 20 mg) recommended as empirical therapy in patients with CAD significantly diminished the severity of angina-like chest pain in 35% of the patients [11]. The present analysis addresses whether such therapy would improve HRQL as well. To our knowledge, this is the first paper concerning this topic.

## Method

Forty-eight consecutive outpatients with CAD-11 female (23%) and 37 male (77%)-diagnosed with recurrent stable angina-like chest pain refractory to standard anti-angina therapy and without indications for revascularization were enrolled in this investigation. The inclusion criteria were as follows: (1) stable angina-like symptoms for at least two months prior to the study recurrent in spite of adequate anti-angina therapy; (2) frequency of chest pain episodes no fewer than three times per week and a frequency of typical heartburn sensation and other gastrointestinal symptoms of no more than once every two weeks due to dietary indiscretion; (3) at least 50% narrowing of the coronary vessels in angiography unsuitable for revascularization in the estimation of an interventional cardiologist; lack of epicardial coronary artery spasm during coronarography; (4) being aged between 40 and 70; and (5) having given written informed consent regarding participation in the study. The exclusion criteria were as follows: (1) lack of consent to study participation; (2) contraindications for carrying out exercise tests; (3) typical symptoms of acute or active chronic disease, including those of the gastrointestinal tract; (4) any change in pharmacological treatment for cardiovascular disease within one month of the start of the study; and (5) the administration of drugs which may affect gastric secretion or digestive tract motility during the month prior to the trial (for example PPIs, H2-receptor antagonists, metoclopramide or cisapride), and/or non-steroidal anti-inflammatory drugs.

### Study protocol

**Figure 1:**
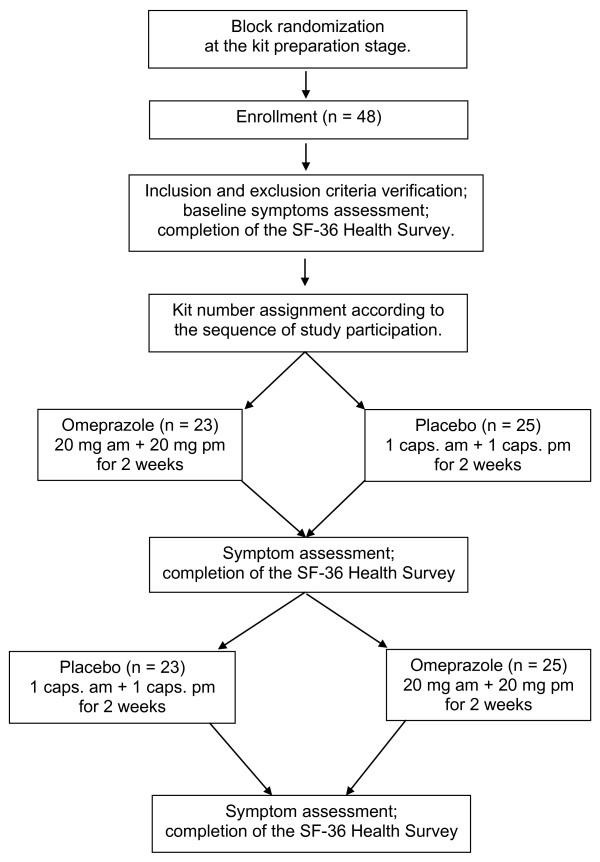
**Diagram of the randomized double-blind, placebo-controlled cross-over study design**.

The full design of this single-center study was a randomized double-blind, placebo-controlled, cross-over investigation (Figure 1) [11,16]. Each patient fulfilling the inclusion and exclusion criteria was first informed of the purpose and principles of the study, as well as given an indication of the prescribed additional drug as being specific to potentially coexistent GER or aspirin-induced gastrointestinal tract damage, and not as therapy for the patient's CAD. None of the patients refused to take part in the investigation. Subsequently, an in-depth interview was carried out with each patient, with special attention paid to baseline angina and gastrointestinal symptom intensity and frequency during the 14 days prior to the start of the study, any therapy undergone and the number of nitroglycerin tablets taken per day thus far, cardiovascular risk factors (hypertension, smoking, alcohol intake, diabetes mellitus, or a family history of cardiovascular events), and any history of coronary interventions. Moreover, each subject was asked to complete the SF-36 Health Survey Standard Polish Version 1.0 9/02 for standard recall (license number: F1-041006-26099). The one modification to this questionnaire consisted of asking the patient to evaluate the two-week period prior to the examination. The original answers obtained to the questions in the SF-36 questionnaire were re-coded and scored using the original 0-100 scoring algorithms and averaged using the respective scale and forms as per the instructions [3]. Three summarized measures were calculated: the total average SF-36 survey score, the physical health component, and the mental health component [3,4]. The first was the sum of all eight health concept scores; the second, known as the physical health component, was the sum of the physical components (role limitations due to personal problems, bodily pain, and general health scale scores); the third arose from summarizing the energy/fatigue (vitality), social functioning, role limitations due to emotional problems, and mental health scale scores.

Following the baseline examination, each patient was assigned to the next consecutive drug kit according to the sequence of his or her participation in this investigation (Figure 1). Each kit consisted of two boxes (A and B) with 28 identical-looking capsules containing either 20 mg of omeprazole or the placebo. According to the random block list generated by the computer at the kit preparation stage, for every ten kits five in box A (given first) contained omeprazole and five the placebo. The list of kit numbers with the respective substance order was inaccessible to the investigator and patients and was stored by the kit producer until the end of the study. In this double-blind fashion, during the first (A) study phase 23 of the subjects obtained omeprazole and 25 the placebo. Within the second (B) phase patients were crossed over to the other arm (omeprazole → placebo or placebo→ omeprazole). A washout period between the two treatment phases was not applied. The patients were asked to take capsules for 14 days, twice a day, 30 minutes before a meal from their respective box. Patients took the first dose of the recommended substance on the evening of the randomization day and the last dose on the morning of the day of the evaluation. In addition, patients continued taking stable doses of previously prescribed drugs (i.e. for CAD, hypertension or diabetes mellitus), including aspirin. Clopidogrel was not recommended for any of the patients. Moreover, no study participant changed smoking habits, alcohol drinking status or lifestyle. The patients were allowed to take short-acting antacids or nitroglycerin as rescue medication and were asked to note such events in a diary.

During the study all the patients were asked to complete the study diary assigned to them. They reported daily the number and severity of chest pain episodes, the circumstances of the appearance of the pain (e.g. whether involving effort, rest, stress or night resting), the necessity for taking nitroglycerin and the number of tablets taken per day, the presence of heartburn episodes and the need to take antacid, the appearance of adverse reactions (with a detailed description), therapy tolerance and the score for their general feeling in accordance with a 10-point scale (similar to a visual analog scale). Moreover, at the end of the investigation phase, the SF-36 questionnaire was completed and a treadmill stress test performed by each patient.

### Ethics

The study protocol was approved by the local Bioethics Committee at the Nicolaus Copernicus University, Collegium Medicum in Bydgoszcz in Poland. All subjects gave their written informed consent prior to their inclusion in the study. All procedures were conducted in compliance with the Declaration of Helsinki.

### Statistics

Statistical analysis was conducted using a licensed version of statistical software STATISTICA PL 9.0 for Windows. Power considerations indicated that a sample size of at least 23 persons was required. The results have been presented as the mean (95% confidence interval or CI) or as a subject number and percentage. Before the analysis, a test for the carryover effect of each health concept defined in the SF-36 survey using a two-stage Grizzle model was performed. The treatment influence was estimated according to intention-to-treat analysis rules. However, due to the lack of a washout period, some doubts as to whether a cross-over design would be appropriate in HRQL studies, and to exclude potential carryover effects on data interpretation, the results presentation has been limited only to those data obtained from the first investigation phase, as in the randomized double-blind, placebo-controlled, parallel study design.

To compare the demographic and clinical data presented in Table 1 Fisher's exact test (for multiway tables) and an unpaired Student t-test were used. The comparisons between groups and study phases were made using one- and two-factorial ANOVA with two repetitions. Fisher's Least Significant Difference (LSD) post hoc test was used to compare the respective values of the SF-36 health concepts after the omeprazole and the placebo phases (Table 2).

**Table 1:** Demographic and clinical data of the studied subjects (n = 48)

Feature	Patients assigned to therapy with omeprazole first (n = 23)	Patients assigned to therapy with the placebo first (n = 25)
Age (years)	58.3 (55.3-61.2)	61.2 (58.1-62.2)

Gender (males, n, %)	19 (83%)	18 (72%)

Number of angina-like symptoms per week (median and range)	10 (6-35)	12 (6-30)

Number of patients with angina-like symptom severity graded according to CCS classification	Class II-19 (83%) Class III-4 (17%)	Class II-17 (68%) Class III-8 (32%)

Dyslipidemia	21 (91%)	24 (96%)

Concentration of total cholesterol (mg/dl)	198.3 (177-220)	197.9 (182.2-213.5)

Concentration of LDL cholesterol (mg/dl)	118.5 (95.6-141.4)	113.1 (103.2-123.0)

Concentration of HDL cholesterol (mg/dl)	52.6 (46.6-58.6)	50.6 (42.5-58.6)

Concentration of triglycerides (mg/dl)	175.8 (125.6-225.9)	176.9 (125-229)

Hypertension	14 (61%)	11 (44%)

Diabetes mellitus	5 (22%)	9 (36%)

Smoking	7 (30%)	5 (20%)

History of myocardial infarction	13 (57%)	17 (68%)

History of PCI	6 (26%)	11 (44%)

History of CABG	3 (13%)	4 (16%)

Ejection fraction in echocardiography (%)	54.5 (47.2-61.7)	53.8 ± 8.4

Aspirin administration	23 (100%)	25 (100%)

Beta-blocker administration	19 (88%)	25 (100%)

Calcium-blocker administration	7 (30%)	6 (24%)

ACEI administration	20 (87%)	19 (76%)

Number of nitroglycerin tablets taken per week (median and range)	3 (0-20)	3 (0-15)

Long-acting nitrate administration	7 (30%)	15 (60%)*

Statin administration	21 (94%)	24 (96%)

BMI (kg/m^2^)	28.4 (26.8-30.0)	28.1 (26.6-29.7)

WHR	0.94 (0.91-0.97)	0.92 (0.89-0.92)

data presented as mean ± 95% CI, or as patient number and %;

*-p < 0.05 between omeprazole and placebo group using Fisher's exact test (for multiway tables) and the unpaired Student t-test

PCI-percutaneous coronary intervention; CABG-coronary artery by-pass graft;

BMI-body mass index; WHR-waist to hip (circumference) ratio;

ACEI-angiotensin-converting enzyme inhibitor.

**Table 2:** The values of the respective SF-36 scores in patients randomly treated with omeprazole and the placebo prior to the study and after two weeks of therapy

SF-36 scales	Omeprazole (n = 23)		Placebo (n = 25)	
	
	At baseline	After 2 weeks	At baseline	After 2 weeks
Total average SF-36 score	53.9 (46.6-61.2)	63.3*# (56.2-70.4)	50.0 (43.7-56.3)	52.9* (45.3-60.6)

Physical functioning	63.5 (54.1-72.9)	68.1 # (57.8-78.3)	56.4 (49.2-63.6)	61.6 (52.9-70.3)

Limitations due to physical health problems	34.8 (15.8-53.7)	59.8# (39.5-80.1)	35.0 (17.4-52.6)	37.0 (20.7-53.4)

Bodily pain	50.5 (39.8-61.3)	66.6*# (55.8-77.5)	41.3 (32.8-49.8)	49.4* (36.2-62.6)

General health perception	45.9 (37.4-54.3)	50.4* (46.2-54.7)	41.0 (34.5-47.5)	43.0* (37.1-49.0)

Physical health component (summarized)	48.7 (38.9-58.4)	61.2*# (52.4-74.4)	43.4 (35.9-50.9)	47.8* (47.3-66.2)

Limitations due to personal and emotional problems	65.2 (46.5-83.9)	75.4 (60.2-90.6)	60.0 (41.4-78.6)	53.3 (34.7-72.0)

Energy/fatigue (vitality)	52.4 (44.7-60.1)	57.6 (49.8-65.4)	46.4 (40.3-52.5)	52.6# (44.4-60.8)

Emotional well-being	52.7 (47.9-57.5)	62.3# (54.8-69.8)	53.9 (49.1-58.8)	57.1 (49.4-64.9)

Social functioning	61.4 (49.8-73.1)	71.2 (60.7-81.7)	64.5 (62.4-76.6)	64.0 (51.3-76.7)

Mental health component (summarized)	57.9 (50.0-65.9)	66.6# (58.8-74.4)	56.2 (48.6-63.8)	56.8 (47.3-66.2)

data presented as mean and 95% CI

*-p < 0.05; statistical significance of difference using Fisher's Least Significant Difference (LSD) post hoc test between omeprazole and placebo groups;

#-statistical significance of difference using Fisher's Least Significant Difference (LSD) post hoc test for comparing the values at baseline and after respective therapy.

## Results

Forty-eight patients were included in the study: 23 randomly assigned to therapy with a double dose of omeprazole for two weeks to be taken first, and 25 to taking the placebo first. The two groups did not differ significantly in the values for their demographic and clinical data (Table 1), the only exception being frequent long-acting nitrate use before the start of the study in patients taking the placebo first (Table 1).

Patients first treated with omeprazole did not differ significantly in baseline SF-36 survey scores in relation to patients assigned to therapy with the placebo (Table 2). In comparison to the values obtained for the two-week period prior to the beginning of the study, the patients treated with omeprazole at the end of the first phase of the investigation in comparison to the subjects taking the placebo had a significantly greater total SF-36 survey score (the sum of all eight of the health concepts), average values for bodily pain, general health perception scales, and physical health summarized into components, being greater by 20%, 35%, 17% and 28% respectively (Table 2).

## Discussion

In this analysis, which is restricted to the first phase originally performed as a double-blind, placebo-controlled cross-over study, it has been shown that the recommendation of a double dose of omeprazole (2 × 20 mg) not only significantly decreased angina-like chest pain occurrence in 17 of the 48 (35%) patients with CAD and the prevalence of some electrocardiographic signs of myocardial ischemia during stress tests, as stated in our previous work [11], but also improved SF-36 scores. Subjects randomly assigned to therapy with omeprazole achieved significantly greater scores than those receiving the placebo in the total SF-36 survey score and for scales concerning a summarized physical health component, especially those of bodily pain and general health perception (Table 2). The greatest relative improvement in SF-36 scores after therapy with omeprazole amounted to 72% (an absolute difference of 25) on average and concerned the scale of limitations due to physical health problems (Table 2). Similar results were shown in the analysis of the full data obtained from the two crossed-over phases of the investigation.

The results obtained could be explained only in part by a decrease in the frequency of acid-related symptom episodes [11]. Observed improvement in HRQL could also have resulted from diminishing activation of the neural cardio-esophageal loop and improvement in myocardial perfusion due to a decrease in esophageal exposure to acid [10,11,17-19]. This is suggested by the greater PPI outcome for physical than for mental health (Table 2). The third explanation for the observed increase in HRQL scores which should be considered is a potential additional decrease in symptoms related to aspirin-induced gastrointestinal tract damage, which may clinically manifest other than as angina-like chest pain. The reported prevalence of this symptom concerned 61% of the patients with CAD [20] and had a major impact on HRQL [4]. However, the high (56%) placebo effect observed in this study, greater than in the work by van Rossum et al. [4] (42%), also suggests some additional role of psychogenic factors in having an impact on observed changes in HRQL scores. Its potential pathway for this effect might be explained by a recently reported omeprazole effect on beta-endorphin plasma level [16].

To our knowledge, the topic of our work has only previously been taken up in the paper by van Rossum et al. [4]. In a double-blind placebo-controlled manner, van Rossum et al. compared the effect of rabeprazole (1 × 20 mg) and a placebo on HRQL as measured by the SF-36 Health Survey in patients with cardiovascular disease requiring therapy with acetylosalicylic acid, both with and without gastrointestinal symptoms, two weeks after Coronary Care Unit discharge. In their study, in contrast to our investigation, rabeprazole was no better than the placebo in the improvement of HRQL relating mainly to gastrointestinal symptom relief. In a multivariate analysis, van Rossum et al. also did not find any influence of clinical data on changes in the summarized physical and mental components of SF-36 scores in responders to rabeprazole (45%), the placebo (42%) and in non-responders, although the first group reported a greater HRQL score than subjects with persistent gastrointestinal symptoms. Apparently similar work by Laheij et al. [15] has been performed according to an observational, non-interventional study design. Its authors, in analyzing the impact of gastrointestinal symptoms on the health status of patients with CAD using the EuroQol (EQ-5D) survey, showed better self-rated health status in patients using drugs to manage gastrointestinal symptoms and complete symptom relief in comparison to subjects who had decided not to be treated with them. Some discrepancies in our study with the results of van Rossum et al. [4] and Laheij et al. [15] might have resulted from differences in the study design, PPI type and doses, patient numbers and inclusion criteria, as our patients did not suffer from clinically manifested gastrointestinal symptoms and symptoms associated with aspirin use were not analyzed.

As mentioned above, only a few papers have been published on the topic of PPI impact on HRQL [4,15] and symptom improvement [11,17] in patients with CAD. However, the favorable effect of PPIs on HRQL, measured using both disease- specific surveys and generic instruments, has been shown in patients without cardiovascular diseases but with upper and lower gastrointestinal symptoms [21], dyspepsia [22], gastroesophageal reflux disease (GERD) [1,23-29], GER-related asthma [30], laryngopharyngeal reflux [31], and for rheumatoid arthritis and arthrosis treated with non-steroidal anti-inflammatory drugs (NSAIDs) [32]. The favorable effect of PPIs on many acid-related diseases was also shown by the exacerbation of GERD symptoms and impairment of HRQL after their discontinuation, probably due to acid rebound hypersecretion [23,33].

Our results seem to have some clinical importance. First, they show the possibility of improving impaired HRQL in patients with CAD through the treatment of coexisting but clinically occult gastrointestinal disorders, not only via a decrease in the severity of symptoms (GER-related chest pain). It was previously reported that HRQL in patients with acid-related disorders is as impaired as that of patients with angina pectoris [34]. Our subjects treated with a double dose of omeprazole obtained an even greater SF-36 score in such health scales as limitations due to personal and emotional problems and the feeling of vitality when these were compared to the mean values reported in the Medical Outcomes Study [6], with the mean values for the physical and mental components determined in healthy Canadian and US populations, as well as in other chronic conditions (e.g. hypertension) [1]. Moreover, the delta of improvement in some components of the SF-36 score after therapy with omeprazole observed in our subjects with CAD was also similar or greater than that seen in recent studies which evaluated the effect of eight months of telephone-delivered collaborative care provided for depressive patients after a coronary artery bypass graft (CABG) [35] and the influence of GERD symptom disappearance on an increase in SF-36 score [1,29,36].

However, it should be underlined that our results cannot be applied in all patients with CAD and refractory angina-like symptoms, mainly because of the questionable but potentially dangerous interaction between omeprazole and anti-platelet drugs. This problem was raised by Juurlink et al. [37], who showed that among patients receiving clopidogrel following acute myocardial infarction, concomitant therapy with PPIs other than pantoprazole was associated with a loss of beneficial effects of clopidogrel and an increased risk of reinfarction. Consequently, many authors have discussed the importance of interactions between PPIs and clopidogrel [38,39] and aspirin [40,41]. However, the conclusion from a recent systematic review by Lima and Brophy is that high quality evidence supporting a clinically significant clopidogrel and PPI interaction is presently lacking [39]. In the recent study on Clopidogrel and the Optimization of Gastrointestinal Events (COGENT) by Bhatt et al. [42], cardiovascular events were also no more common with omeprazole, but Juurlink [43] supports the greater safety of pantoprazole.

Our study has some limitations. Firstly, the sample size was too low but this did not prevent the reaching of statistical significance in many of the comparisons (Table 2). Secondly, the patients were not investigated gastroenterologically, whereas e.g. GERD type (non-erosive or erosive) as well as a diagnosis of *Helicobacter pylori *infection might affect the PPI therapy outcome [26,27]. However, empirical therapy with PPIs is an approved gastroenterological diagnostic tool for GER-related symptoms, including GER-related chest pain [14,44], and responders to PPIs do not need an additional examination if they do not present warning symptoms. Van Rossum et al. [4] and Laheij et al. [15] did not examine their patients gastroenterologically either. Thirdly, the study analysis was performed in a way other than originally planned but, in our opinion, this can be justified by the intention to avoid bias due to carryover effects and to allow a clearer presentation of the results. Fourthly, patients randomly assigned to therapy with omeprazole had only occasionally been treated with long-acting nitrates (Table 1). This difference may have affected the investigation outcomes in different ways. On the one hand, long-acting nitrates are recommended in patients with greater severity of symptoms. If the symptoms had been cardiac in origin rather than misdiagnosed GER-related chest pain, the potential effect of PPIs on symptom severity and HRQL score might have been less. On the other hand, long-acting nitrates reduce the severity of angina pectoris and can induce GER, giving greater probability to achieving a better self-rated health status. Fifthly, the study was performed with gastric acid secretion inhibitors and was oriented towards a decrease in acid-related symptom severity, but specific HRQL questionnaires, e.g. Quality of Life in Reflux and Dyspepsia, were not used. However, this investigation was performed with patients with CAD and the gastroenterological origin of symptoms was not clinically overt. Therefore, similar to the investigation by van Rossum et al. [4], the greater usefulness of general measures such as the SF-36 survey was assumed. This commonly used instrument allows comparisons across conditions and interventions. Moreover, the assumed inclusion criteria for patients without severe gastrointestinal manifestation could not have enabled the demonstration of a significant effect of omeprazole with the use of a questionnaire oriented to acid-related disorders. The correction of the decision to use a generic HRQL survey also justified the statistical significance obtained for the differences in this small study sample. In our opinion, this method demonstrated that the disadvantages of using the SF-36 survey were fewer than the advantages.

In conclusion, a double dose of omeprazole improved the general HRQL in patients with stable CAD who were without severe gastrointestinal symptoms more effectively than the placebo.

## List of abbreviations

HRQL: health-related quality of life; CAD: coronary artery disease; GER: gastroesophageal reflux; PPI: proton pump inhibitors; HRQLRAD: Quality of Life in Reflux and Dyspepsia questionnaire; AF: HRQL: Quality of Life questionnaire for patients with atrial fibrillation.

## Competing interests

The authors declare that they have no competing interests.

## Authors' contributions

JB prepared the manuscript and performed the statistical analysis. JB, GP, KS, JF, MM, MK, BGP and MW designed the study protocol, organized and carried out the original study, and took part in the acquisition of data and their analysis and interpretation. All authors read and approved the final manuscript.
